# Clinical Prognosis of Right-Sided Infective Endocarditis not Associated with Cardiac Devices or Intravenous Drug use: a Cohort Study and Meta-Analysis

**DOI:** 10.1038/s41598-020-64220-z

**Published:** 2020-04-28

**Authors:** Pau Vilardell Rigau, Sergio Moral, Daniel Bosch, Manel Morales, Josep Maria Frigola, Xavier Albert, Rocío Robles, Esther Ballesteros, Marta Roqué, Jaime Aboal, Ramon Brugada

**Affiliations:** 1 Cardiology Department, Hospital Universitari Doctor Josep Trueta, CIBER-CV, Girona, Spain; 2 Radiology Department, Centre d´Atenció Primaria Pare Claret, Institut Català de la Salut, Barcelona, Spain; 3 Iberoamerican Cochrane Centre, Biomedical Research Institute Sant Pau (IIB Sant Pau), CIBER Epidemiología y Salud Pública (CIBERESP), Barcelona, Spain

**Keywords:** Cardiovascular biology, Valvular disease

## Abstract

Right-sided infective endocarditis (RSIE), classically associated with intravenous drug use or intracardiac devices, is considered a good-prognosis infective endocarditis (IE) form. However, predisposing factors and prognosis for “NODID” RSIE (**NO**t associated with cardiac **D**evices or **I**ntravenous **D**rug use) remain unclear. The aim of this study was to evaluate predisposing factors and prognosis of NODID RSIE compared to other RSIE forms. A retrospective cohort study (January 2008–January 2019) was conducted in a reference center on 300 patients diagnosed with IE. Endocarditis-related events were defined as related to IE in mortality or open-heart surgery during follow-up. A review and meta-analysis of associated literature (January 2008-January 2019) were also performed. Fifty-seven patients presented RSIE (19%), 22 of which were NODID RSIE (39%). Use of intravascular catheters (23% vs 3%; *p* = *0.027*) and congenital heart diseases (18% vs 0%; *p* = *0.019*) were associated with NODID RSIE. This group had a higher in-hospital mortality (23% vs 3%; *p* = 0.027) and endocarditis-related event rates (41% vs 6%; *p* = 0.001) than non-NODID RSIE. Furthermore, NODID RSIE was independently associated with in-hospital endocarditis-related events (OR = 19.29; 95%CI:2.23–167.16; *p* = 0.007). Our meta-analysis evaluated four studies and identified 96 cases (30%) of NODID RSIE from 320 total RSIE cases. NODID RSIE patients demonstrated higher in-hospital mortality (RR = 2.81; 95%CI:1.61–4.90; *p* < 0.001; I^2^ = 0.0%) and necessity of open-heart surgery (RR = 13.89; 95%CI:4.14–46.60; *p* < 0.001; I^2^ = 0.0%) than non-NODID RSIE cases. Our study suggests that NODID RSIE has the highest endocarditis-related event rate and in-hospital mortality among RSIE cases and therefore should not be considered a good-prognosis IE.

## Introduction

Right-sided infective endocarditis (RSIE) is considered a good-prognosis form of infective endocarditis (IE), with in-hospital mortality of 5–10%^1–4^. This pathology is classically associated with intravenous drug use (IVDU) or intracardiac devices (pacemakers or defibrillators). Recommended treatment is antimicrobial therapy and complete hardware removal in cases associated with intracardiac devices, but rarely open-heart surgery^1,5,6^.

The benign in-hospital course of RSIE is mainly based on youth and low comorbidities of IVDU cases and good results from combined antimicrobial treatment and hardware removal in patients with cardiac devices^7–10^. Nevertheless, in the past decade a new group of RSIE not associated with cardiac devices or IVDU (NODID) has been described,but its prognosis and predisposing factors are not wellestablished^9,11–14^. Some authors have indicated that these patients may have a worse disease course, raising the question of a potential need fornew therapeutic approachesinthese cases^11^. However, there is currently no consensuson the prognosis and best treatment option for NODID RSIE.

Therefore, this study determined the clinical evolution of NODID RSIE in a recent cohort of IE patients and established thepossible predisposing factors for this group. Further, we conducted a systematic review and meta-analysis of recently published studies to evaluate the global prognosis of NODID RSIE.

## Methods

### Study population

**Figure 1:**
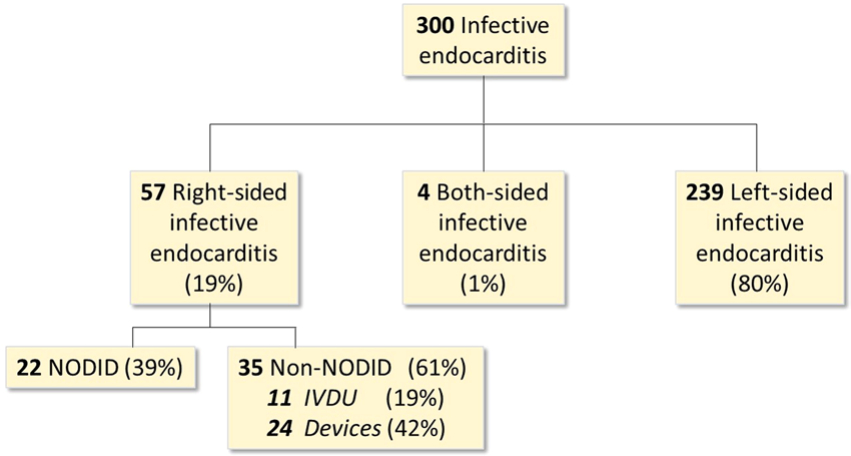
The overall cohort of infective endocarditis. IVDU = Intravenous drug users.

From January 2008–January 2019, 300consecutive patients diagnosed with IE were retrospectively included in this study. Of these 300 patients, 57were diagnosed with RSIE (Fig. 1) according to modified Duke criteria^1,15^. Patients were excluded if they had concomitant left-sided infective endocarditis (LSIE) or an unclear diagnosis. All patients underwent transthoracic echocardiography and transesophageal echocardiography and/or positron emission tomography–computed tomography if required. NODID RSIE was defined asRSIE patients without intracardiac devices or IVDU history. Those patients which we could not confirm this data were excluded. The protocol received institutional review board approval by University Doctor JosepTrueta hospital ethics committee. The informed consent was waived due to the retrospective nature of the study according to the ethics committee approval and all tests were performed in accordance with relevant guidelines and regulations.

### Baseline measures and follow-up

Baseline clinical, microbiology, imaging, and medical and invasive treatment data were obtained from medical records. Medical and surgical treatment was determined by the endocarditis team of the institution, per guidelines and each case^1,2^. Endocarditis-related events were defined as related to IE in mortality or open-heart surgery. Removal of cardiac devices was not considered open-heart surgery. Pulmonary and systemic embolisms were also collected. Malnourished patients were defined using the Mini Nutritional Assessment Short Form (MNA-SF)^16^.

After discharge, retrospective follow-up was performed by medical record review or telephone contact. Causes of death were defined according to medical records and death certificates. Follow-up time was defined as number of months between the event and first endpoint event; the most recent outpatient visit or telephone contact was considered the end of follow-up for patients who did not reach an endpoint.

### Systematic review and meta-analysis

**Figure 2:**
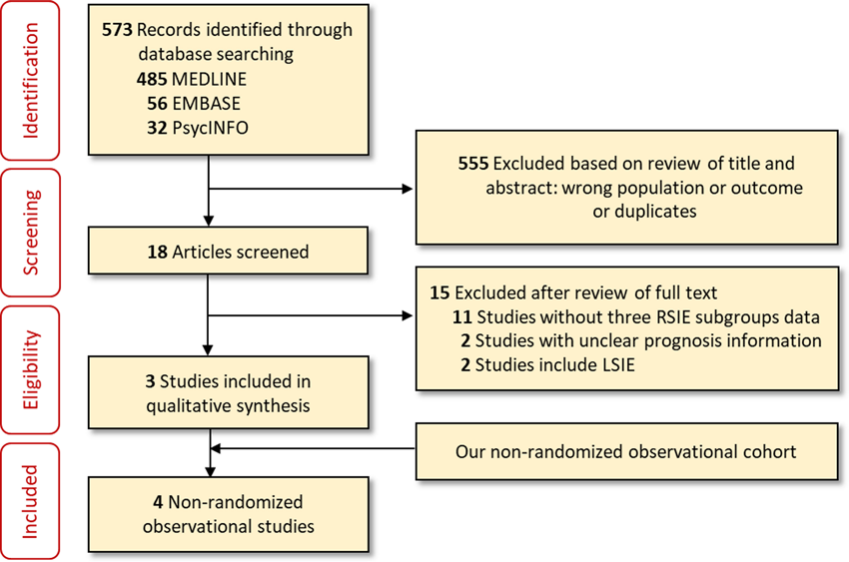
Flow diagram of trial selection.

Studies reported during January 2008–January 2019that included RSIE were identified with EMBASE, MEDLINE, and PsycINFOsearches (independently performed by P.V., S.M., and E.B.) by screening references of identified articles and by correspondence with study researchers using the approach recommended by PreferredReporting Items for Systematic Reviews and Meta-Analyses (PRISMA) guidelines(Fig. 2)^17^. Computer-based searches combined terms related to RSIE andright heart cavities with different synonyms in the medical literature (full details of the search strategyare provided inSupplementary Table A1). Studieswere included if they had a cohort design, reported data on the prognosisof NODID and non-NODIDRSIE casesand were published in peer-reviewed journals. NODIDRSIE cases were definedas those without association to IVDU or cardiac devices. Studies without clear definition of NODIDRSIE orwith < 15 patients were excluded. Case-controlstudies were also excluded.

Data extraction and quality assessment were conducted independently by two

investigators (P.V. and S.M.), and all discrepancies were resolved by discussion andadjudication of a third reviewer (E.B.). The following data were extracted using a standardizedform: study design, geographic location, sample size, average age of participants,percentage of male participants, number of cases with RSIE, number of patientswith NODID and non-NODID RSIE at diagnosis, endocarditis mortality, andopen-heart surgery related to IE.

The Newcastle-Ottawa Scale(NOS) was used to assess quality of cohort studies^18^. Thisscale assesses sample representativeness, comparabilitybetween patients with and without NODID RSIE, quality of outcome assessment, andadequacy of follow-up (full scoring details in Supplementary Appendix A1). Risk of biaswas deemed high if a study scored 0–3, moderate if a study scored 4–6, andlow if a study scored7–9^19^.

Primary endpoint of the meta-analytic study was mortality secondary to RSIE during hospitalization,regardless of treatment. Secondary endpoint was in-hospital open-heart surgery related to IE.

### Statistical analysis and data synthesis

SPSS version 17.0 software (IBM Inc., New York, New York) and Review Manager 5.3 software and STATA version 11.2 (StataCorp, College Station, Texas) were used for statistical computations. Descriptive data are presented as mean±SD, medians (interquartile range), or proportions, depending on variable distribution. For continuous variables, deviations from normality were determined with the Kolmogorov-Smirnov test. For categorical variables, significant differences between groups were assessed with chi-square or Fisher exact tests. Differences among groups for continuous parameters were assessed by Student’s t test or analysis of variance with Bonferroni correction for multiple comparisons if normally distributed, and Mann-Whitney U or Kruskal-Wallis tests if not normally distributed. A *p*-value of < 0.05 was considered significant. Univariable logistic regression was performed to identify determinants of endocarditis-related events. Factors significant at the *p* < 0.10 level in the univariate analyses were included in multivariable logistic regression analysis. Indicators with a 95% confidence interval for the odds ratio (OR) not including 1 were considered significant.

Meta-analyses were conducted to compare prognoses between NODID and non-NODIDRSIE cases for primary and secondary endpoints. Prognosis was measured with relative risks (RR) with 95% confidence intervals (CI). Pooled RR was computed for primary and secondary endpoints with the DerSimonian-Laird method in a random effects model. Heterogeneity among studies was assessed qualitatively and quantitatively (using chi-square test of heterogeneity and I2 statistic).

## Results

### Baseline characteristics of study population

In a cohort of 300 IE patients, 57patients (19%) were diagnosed with RSIE: 22NODID(39%) and 35 non-NODID(61%) cases, including 11 non-NODIDcases(19%) with IVDU and 24(42%) with cardiac devices(Fig. 1). Baseline characteristics of NODIDand non-NODIDRSIE cases are detailed in Table 1.

**Table 1:** Demographic, clinical, microbiological, and imaging characteristics of NODID and non-NODID right-sided infective endocarditis (RSIE) cases.

Variable (n = 57)	Non-NODID RSIE (n = 35)	NODIDRSIE (n = 22)	*p*
**Gender [males; n (%)]**	24 (69)	15 (68)	0.975
**Age (years; mean** ± **SD)**	60.3 ± 19.7	56.7 ± 18.9	0.500
**COMORBIDITIES**			
**Hypertension, n (%)**	17 (49)	8 (36)	0.366
**Dyslipidemia (%)**	13 (37)	6 (27)	0.442
**Diabetes mellitus, n (%)**	9 (26)	6 (27)	0.897
**HIV, n (%)**	5 (14)	0 (0)	0.145
**Hemodialysis, n (%)**	1 (3)	3 (14)	0.288
**Cancer, n (%)**	2 (6)	4 (18)	0.192
**Intravascular catheter, n (%)**	**1 (3)**	**5 (23)**	**0.027**
**Coronary heart disease, n (%)**	8 (23)	4 (18)	0.750
**Left-side prosthetic or repaired valve, n (%)**	1 (3)	3 (14)	0.288
**Right-side prosthetic or repaired valve, n (%)**	1 (3)	1 (5)	>0.999
**Congenital heart disease, n (%)**	**0 (0)**	**4 (18)**	**0.019**
**Charlson score (mean** ± **SD)**	2.0 ± 1.6	2.3 ± 1.6	0.536
**MICROBIOLOGY**			
***Staphylococcusaureus*, n (%)**	**22 (63)**	**6 (27)**	**0.009**
***Staphylococcusepidermidis*, n (%)**	3 (9)	2 (9)	>0.999
***Enterococcus faecalis*, n (%)**	2 (6)	1 (5)	>0.999
***Streptococcusviridans*, n (%)**	0 (0)	2 (9)	0.145
***Streptococcusbovis/gallolyticus*, n (%)**	1 (3)	1 (5)	>0.999
***Escherichiacoli*, n (%)**	1 (3)	0 (0)	>0.999
***Pseudomonas aeruginosa*, n (%)**	0 (0)	1 (5)	0.386
***Staphylococcuscapitis*, n (%)**	0 (0)	1 (5)	0.386
***Streptococcusmitis*, n (%)**	0 (0)	1 (5)	0.386
**Other microorganisms, n (%)**	5 (14)	4 (18)	0.722
**Multiple microorganisms, n (%)**	0 (0)	2 (9)	0.145
**Negative, n (%)**	1 (3)	1 (5)	>0.999
**CLINICAL PRESENTATION**			
**Fever, n (%)**	33 (94)	21 (96)	>0.999
**Dyspnea, n (%)**	5 (14)	7 (32)	0.114
**Hemoglobin (mg/dL), median (IQR)**	11.1 (3.2)	10.7 (2.4)	0.142
**Hematocrit, mean** ± **SD**	34.2 ± 7.3	32.6 ± 4.5	0.383
**Leucocytes (10**^**6**^**/L), mean** ± **SD**	11527 ± 6531	12460 ± 6828	0.608
**CRP (mg/dL), mean** ± **SD**	10.2 ± 7.9	12.8 ± 11.1	0.311
**Creatinine (mg/dL), median (IQR)**	0.8 (0.62)	1.0 (0.82)	0.456
**Protein (mg/dL), mean** ± **SD**	6.3 ± 1.2	6.2 ± 1.0	0.701
**Albumin (mg/dL), mean** ± **SD**	3.4 ± 0.9	3.4 ± 0.8	0.869
**Prealbumin (mg/dL), mean** ± **SD**	20.9 ± 7.6	19.8 ± 6.8	0.577
**Nutritional status (malnourished), n(%)**	14 (40)	10 (46)	0.685
**Kinds of antibiotics used per patient, median (IQR)**	2 (1)	2 (1)	0.748
**ECHOCARDIOGRAPHIC FINDINGS AT DIAGNOSIS**			
**Location of major vegetation**^**a**^**, n (%)**			
Tricuspid valve	10 (91)	13 (59)	0.123
Pulmonary valve	0 (0)	6 (27)	
Other locations	1 (9)	3 (14)	
**Morphological complication**^**b**^**, n (%)**			0.288
No	34 (97)	19 (86)	
Yes	1 (3)	3 (14)	
**Major diameter of the vegetation (mm), mean** ± **SD**	15.5 ± 8.5	18.4 ± 10.7	0.279
**LVEF (%), median (IQR)**	58.0 (10.0)	58.5 (10.5)	0.948
**TAPSE (mm), mean** ± **SD**	20.3 ± 3.7	20.6 ± 3.4	0.777
**FAC (%), median (IQR)**	44.7 (10.0)	43.1 (7.3)	0.470
**Significant TR (** ≥ **3), n (%)**			0.614
**No**	23 (66)	13 (59)	
**Yes**	12 (34)	9 (41)	
**Significant PR (** ≥ **3), n (%)**			
**No**	35 (100)	19 (86)	0.053
**Yes**	0 (0)	3 (14)	

^a^Excluding RSIE with intracardiac devices.

^b^Including perforation, fistulae, and abscess.

HIV = human immunodeficiency virus; CRP = C-reactive protein; LVEF = left ventricular ejection fraction; TAPSE = tricuspid annular plane systolic excursion; FAC = fractional area change; TR = tricuspid regurgitation; PR = pulmonary regurgitation; IQR = interquartile range.

Compared to non-NODID RSIE patients, NODID RSIE cases included a significantly higher percentage of intravascular catheter carriers (23% vs 3%; *p* = 0.027) andcongenital heart diseaseat baseline (18% vs 0%; *p* = 0.019). Etiology for NODID RSIE was mainlypolymicrobial,while non-NODIDRSIE casespresented *Staphylococcusaureus*as the main etiopathogen (63%) compared to NODID RSIE cases(27%; *p* = 0.009). NODID and non-NODIDRSIE groups did not differ in clinical presentation and echocardiogram findings (Table 1).

### Clinical prognosis by RSIE subgroup

Of the 57 RSIE cases, 6 patients (11%) died during hospitalization, all secondary to IE, and 5 of these patients(83%) hadNODID RSIE. Furthermore, 5of the 57 RSIE patients(9%) required open-heart surgery:2 patients had tricuspid valve replacement, 2 patients had tricuspid valve repair with vegetation removal, and 1 case had vegetation removal and ventricular septal defect repair. Of those 5 patients, 4 (80%) had NODID RSIE and 1 (20%)was associated with IVDU. No patients who underwent open-heart surgery died during hospital admission. Mean hospital stay was: 24.7 ± 20.0days (range:2–122 days; median: 23 days; quartiles 1–3: 11–32 days). NODID RSIE patientshad worse prognosis than non-NODID cases, based on rate of adverse endocarditis-related events during hospitalization (41% vs 6%; *p* = 0.001) and in-hospital mortality (23% vs 3%; *p* = 0.027)(Table 2). Additionally,NODID RSIE cases had ahigherrate of open-heart surgery than non-NODID cases (18% vs 3%; *p* = 0.067).

**Table 2:** Clinical complications of patients with NODIDor non-NODID right-sided infective endocarditis(RSIE) during hospitalization and after discharge.

Variable	Non-NODID RSIE	NODID RSIE	*p*
**CLINICAL OUTCOME DURING HOSPITALIZATION**			
(n = 57)	(n = 35)	(n = 22)	
**In-hospital mortality and/or open-heart surgery**	**2 (6)**	**9 (41)**	**0.001**
**In-hospital mortality**	**1 (3)**	**5 (23)**	**0.027**
**Open-heart surgery**	1 (3)	4 (18)	0.067
**Pulmonary embolism**	17 (49)	11 (50)	0.916
**Systemic embolism**	3 (9)	0 (0)	0.276
**CLINICAL OUTCOME AFTER DISCHARGE**			
(n = 51)	(n = 34)	(n = 17)	
**Mortality for any cause of death**	7 (21)	6 (35)	0.256
**Mortality for oncologic pathologies**	0 (0)	2 (12)	0.107
**Mortality for respiratory pathologies**	1 (3)	1 (6)	>0.999
**Mortality for cardiac pathologies**	1 (3)	2 (12)	0.255
**Mortality for right heart failure**	0 (0)	1 (6)	0.333
**Mortality for other causes**	5 (15)	1 (6)	0.650
**Relapse**	1 (3)	0 (0)	>0.999

Values aren (%).

IVDU = Intravenous drug users; RSIE: right-sided infective endocarditis.

Fifty-one patients were discharged after treatment. Follow-up ranged 1–127 months (mean: 49 ± 41 months; median: 35 months; quartiles 1–3: 12–83 months). Of these 51 patients, 13 patients (25%) died during follow-up: 12 from non-RSIE endocarditis complications (2 from neoplasia, 2 from respiratory infection, 4 from multiorgan failure due to sepsis, 2 from left-sided heart failure decompensation, 1 from acute renal failure, and 1 from complicated femur fracture), and 1 from right-sided heart failure decompensationwith chronic right ventricle dysfunction (this patient had NODID RSIE). No differences were found during follow-up in mortality or morbidity between NODIDor non-NODID RSIEcases (Table 2). No patients required open-heart surgery during follow-up, andonly 1 non-NODID RSIE patient presented relapse.

In the subgroup analysis dividing the non-NODID group in IVDU and cardiac devices carriers the results during hospitalization and follow-up were similar than between NODID and non-NODID groups, with the exception of pulmonary and paradoxical systemic embolisms, which were more frequent in the IVDU group (Supplementary Table A2). The subanalysis evaluating NODID group separately (congenital heart disease patients vsintravascular catheter carriers vspatients without these features) showed that these subgroups presented worse prognosis than RSIE associated with cardiac devices but the worse prognosis for congenital heart disease group was secondary to the high rate of interventions (50% of cases; Supplementary Table A3).

### Predictors of adverse endocarditis-related events for RSIE

Endocarditis-related event rate (in-hospital mortality or open-heart surgery during IE event) was significantly higher in patients with C-reactive protein (CRP) levels >16 mg/dL(45% vs 13%; *p* = 0.022), patients undergoing hemodialysis (27% vs 2%; *p* = 0.020) and patients withCharlson score >3 (45% vs 17%; *p* = 0.056). Neither *Staphylococcus aureus* (14% vs 24%; p = 0.346) nor polymicrobial infections (50% vs 18%; p = 0.352) were significantly associated with endocaditis-related event rate in our series. NODID RSIE caseswere related to adverse events: 41%(9 of 22 cases) died (n = 5) or required open-heart surgery (n = 4) during hospitalization (*p* = 0.004). Only NODIDRSIE cases(OR = 19.29; 95%CI: 2.23–167.16; *p* = 0.007)and those withCharlson score >3 (OR = 9.75; 95%CI: 1.30–73.17; *p* = 0.027) were associated with endocarditis complications during hospitalization. A nonsignificant trend for endocarditis-related events was also observed in patients withCRP > 16 mg/dL (OR = 7.35; 95%CI: 0.78–69.40; *p* = 0.082).

### Meta-analysis of RSIE clinical prognosis

Our initial search identified 573 publications. After screening titles and abstracts, 18 publications were selected for full-text review; of these, 15did not meet inclusion criteria (Fig. 2). Therefore, we combined 3 published studies^10,11,13^ with our results (1 longitudinal prospective and 3 longitudinal retrospective cohorts) for a meta-analysis involving a total of 320 individuals with RSIE.

**Figure 3:**
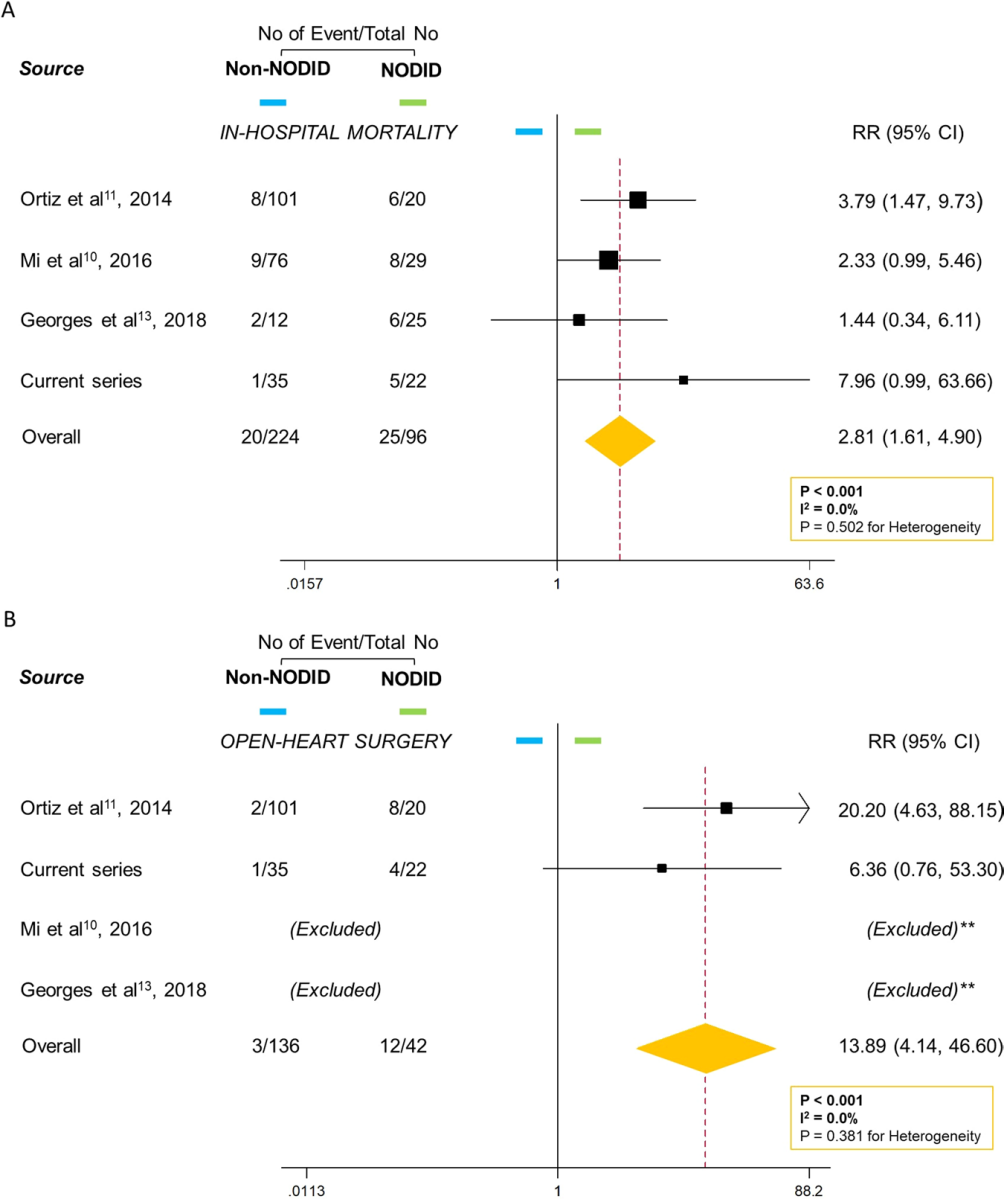
Rates of (**A**) in-hospital mortality and (**B**) open-heart surgery for NODID versus non-NODID right-sided infective endocarditis (RSIE) cases during hospitalization from four studies. Boxes are proportional to weight of each study in the analysis, and lines represent 95% confidence intervals (CI). Open diamond represents pooled relative risk, and its width represents 95% CI. **Studies without events in both groups or lack of information are excluded.

EstimatedNODID RSIE incidence for the 4 studies was 30% (96 of 320 individuals; range: 17%–68%),with in-hospital mortality of 26% (25 of 96 individuals; range: 23%–30%). Main characteristics of each study cohort are reported in Supplementary Table A4. Risk of bias among the included studies was low (mean NOS score: 8 of 9; Supplementary Table A5). Endocarditis-related mortality during hospitalization occurred in 45 patients (14%). Open-heart surgery could only be assessed in 2 studies, which indicated an incidence of 8% (15 of 178 individuals). NODID RSIE cases presented higher in-hospital mortality (RR = 2.81; 95%CI: 1.61–4.90; *p* < 0.001; I^2^ = 0.0%; Fig. 3A) and higher necessity of open-heart surgery (RR = 13.89; 95%CI: 4.14–46.60; *p* < 0.001; I^2^ = 0.0%; Fig. 3B) than non-NODID RSIE cases.

## Discussion

This study demonstrates that NODID RSIE, without association to IVDU or cardiac devices, has the worst clinical prognosis among RSIEwith in-hospital mortality of >20%. This poor prognosis was also observed in meta-analysis of published literature,and NODIDRSIE patients had a higher rate of open-heart surgerythanother RSIE cases. Additionally, predisposing factors for NODID RSIE also differ,as intravascular catheters and congenital heart disease were more frequent in these cases.

### Incidence and predisposing factors for NODID RSIE

Incidence of RSIE has been described as 5%–10% of IE^1^. However, recently published studies indicate an increased incidence of 10%–20%^8,11,14,20^. Our study found a similarly increased incidence of 19%. These changes may reflect growing predisposing risk factors for RSIE, especially for NODID cases.

Developed countries have recently experienced an increasedpopulation with comorbiditiesthat requiremore invasive explorations and venous access, as carriers of intravascular cathetersparticularly affectRSIE incidence^1,3^. Georges *et al*.^13^ reported intravascular cathetersare a portal entry for RSIE in 27% of cases from an intensive care unit cohort. Furthermore,Chrissoheris *et al*.^21^ reported right heart cavities are affected in 67% of cases with IE attributed to intravascular catheters. In addition, congenital heart disease in adults, usually associated with RSIE,is currently more prevalent thanit was 10 years ago due to improvedtreatments, especially in children^1,3,22^. Ruotsalainen *et al*.^23^ reported five-times greater risk of*Staphylococcusaureus*IE in patients with bacteremia and congenital heart disease than in those withoutthese conditions.

Otherfactorssuch as hemodialysis treatment orprevious cancer seem to berisks for NODID RSIE^3,11,21^, although there is a tendency, in our study these associationswere not significant. A subanalysis by Ortiz *et al*.^11^ described a significant association of NODID RSIEwith cancer and chronic renal failure. Further, Ludvigsen *et al*.^24^ reportedIE incidence is38-times higher for hemodialysispatients than untreated patients. Theseepidemiological changes could explain not only the increase in numbers, but also the increase in RSIE cases compared to LSIE in our IE population, especially for NODIDRSIE patients^11,14,20^. In a nutshell, patients on hemodialysis, congenital heart disease, with polymicrobial infections or malignancy have a higher mortality rate when being treated for RSIE.

### Prognosis of RSIE

Mortality of RSIE patients has been mainly analyzed based on IVDU and cardiac device use^1^. In our study, NODID RSIE carried a high risk of endocarditis-related complications, with in-hospital mortality similar toLSIE, which is ~20%–30%^25^. This higher mortality was also confirmed in our meta-analysis, showingglobal worse prognosis of RSIE patients. This group also hada higher rate of open-heart surgerythannon-NODID RSIE cases, although those who underwent surgery did not have a worse prognosis and were all discharged after theintervention. Other studies have demonstrated good results for open-heart surgery on isolated RSIE,with in-hospital mortality of <6%^26^. Nevertheless, open-heart surgery rates for LSIE, usually between ~35%–60%, are significantly higher thanfor RSIE, even forNODID RSIE with similar complexity scores^5,25^.

Further, Charlson score also was a strong independent factor for RSIE-related complications, highlighting a highrisk of complications forNODIDRSIE caseswith Charlson score >3. In addition, CRP is a laboratory riskmarker that is well-studied in IE^27,28^. Our univariate analysis demonstrated a relationship between CRP > 16 mg/dL and endocarditis complications. However,it is well-known as unspecific biomarker andfurther studies are needed to confirm CRP as a possible tool for making treatment decisions.

Ramos-Martínez *et al*.^29^ demonstrated significantly higher in-hospital mortality for patients undergoing hemodialysis compared to untreated patients. Althoughthe cohort included RSIE and LSIE, our results are concordantwith these data and indicate that patients with RSIE and undergoing hemodialysis could have a worse prognosis.

### Clinical implications of pure RSIE

**Figure 4:**
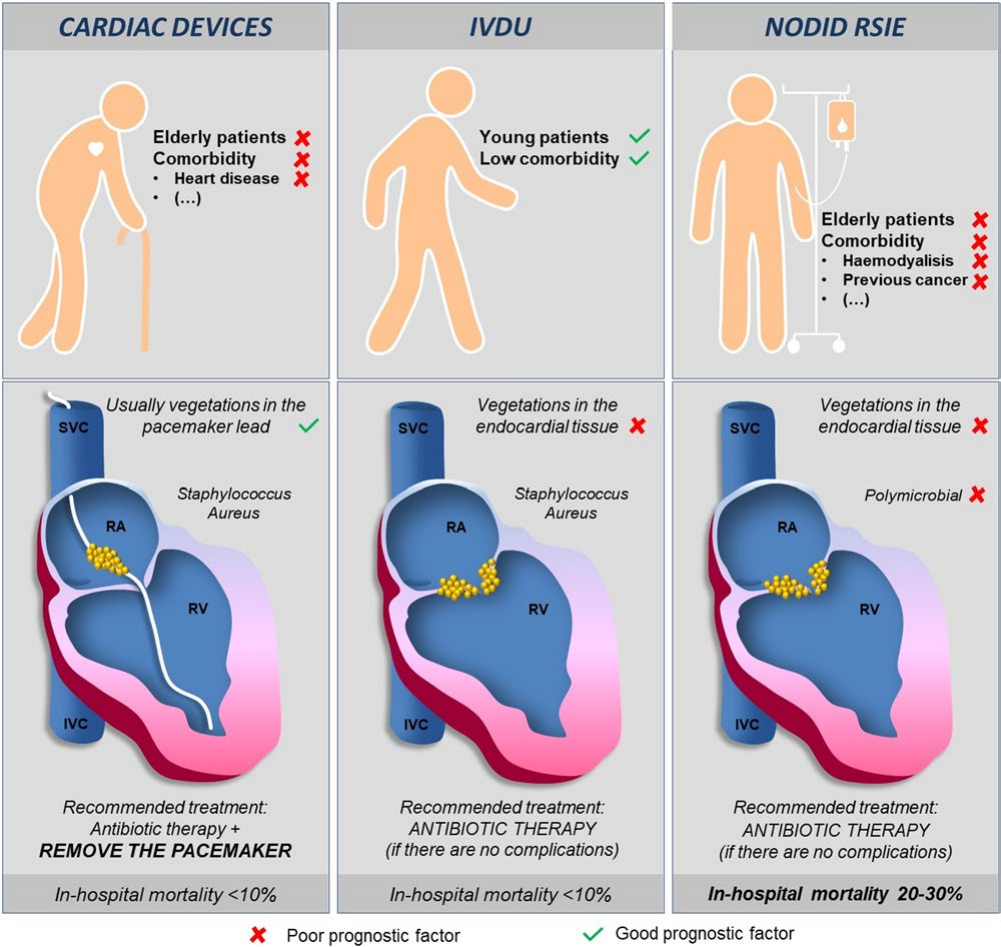
Main differences in basal demographic, clinical, microbiological and imaging characteristics according to the RSIE group (NODID, IVDU and cardiac devices carriers) which may explain the worst prognosis of NODID cases with the recommended treatment.

NODID RSIE is a group with a poor prognosis,similar to LSIE. Therefore, our results break the good-prognosis paradigm that has been described for RSIE and should alter how NODIDRSIE is preventedand treated. Based on our findings, we may hypothesize that demographic, clinical, microbiological and imaging characteristics in NODID versus non-NODID cases may differ and therefore the response to a similar treatment is also different (Fig. 4). Preventive programs in high-risk groups, including intravascular catheter carriers, chronic hemodialysis patients, or cases with congenital heart disease, should be updated to decrease the potential number of new RSIE cases. Further, high in-hospital mortality of these patients may change the treatment decision-making scheme classically accepted for RSIE. Invasive treatment guidelines and expert consensus of IE are usually wider for LSIE than RSIE^1,2,5^. However, as our results and other studies show, in-hospital surgery has good results in RSIE mortality rate^26,30^. For this reason, indications of open-heart surgery in RSIE should be reviewed, especially in cases ofNODID RSIE with other markers of complicated course, such as Charlson index >3,because of the high risk of in-hospital mortality for these patients. As in LSIE, early surgery in high-risk cases without contraindications could improve therapeutic results in those patients^1,2,5^. Furthermore, a new percutaneous approach proposed for tricuspid vegetation removal in high-risk surgical caseshas shown good results;this may provide another therapeutic solution forpatients with NODID RSIE^31^. Nevertheless, further studies are required to confirm our data.

### Study limitations

We are aware of several limitations of our work. First, the retrospective study design and unicentricdata source used affects to underestimation of the true number of IE cases. Second, selection bias inherent to the tertiary hospital with cardiac surgery department also affects the number of IE. Third, although our study showsthat NODID RSIE is associated with in-hospital endocarditis-related events and mortality, statistical analysis does not include all possible influential prognosis variables. Nonetheless, we were able to analyze more than 20 of these variables that demonstrate our findings. Furthermore, our meta-analysisis based on only four longitudinal studies and three of them are retrospective, sothe results are limited. Nevertheless, this analysis is the largest series evaluating NODID RSIE prognosis and thus provides information on the global tendency of this group in the past 10 years. Larger studies are required to identify possible therapeutic optionsto decrease mortality of thesepatients. All these limitations should be taken into account when interpreting our results.

## Conclusions

Our study suggests that NODID RSIE,not associated with cardiac devices or IVDU, has a poor in-hospital prognosis and higher necessity for open-heart surgery than other RSIE groups. Additionally, predisposing factors such as intravascular catheters or congenital heart disease should be evaluated as risk factors to identify new approaches to prevent NODID RSIE.

## Supplementary information

Supplemental Material.
