# Supplementary material for: Clinical Prognosis of Right-Sided Infective Endocarditis not Associated with Cardiac Devices or Intravenous Drug use: a Cohort Study and Meta-Analysis
Source: Sci Rep. 2020 Apr 28;10:7179. doi: 10.1038/s41598-020-64220-z (PMC7188839; doi:10.1038/s41598-020-64220-z)
Supplement: Supplementary file 1 — Supplemental Material. [file 41598_2020_64220_MOESM1_ESM.doc]

# SUPPLEMENTARY MATERIAL

Article Title:

CLINICAL PROGNOSIS OF RIGHT-SIDED INFECTIVE ENDOCARDITIS NOT ASSOCIATED WITH CARDIAC DEVICES OR INTRAVENOUS DRUG USE: A COHORT STUDY AND META-ANALYSIS

**List of Authors and Their Academic Degrees:** Pau Vilardell, MD (1), Sergio Moral, MD, PhD (1), Daniel Bosch, MD (1), Manel Morales, MD (1), Josep Maria Frigola, MD (1), Xavier Albert, MD (1), Rocío Robles, MD (1), Esther Ballesteros, MD (2), Marta Roqué, BSc (3), Jaime Aboal, MD (1), Ramon Brugada, MD, PhD (1).

**List of Authors’ Affiliations:**

1. Cardiology Department, Hospital Universitari Doctor Josep Trueta, CIBER-CV (Girona, Spain)
2. Radiology Department, Centre d´Atenció Primaria Pare Claret, Institut Català de la Salut (Barcelona, Spain)
3. Iberoamerican Cochrane Centre, Biomedical Research Institute Sant Pau (IIB Sant Pau), CIBER Epidemiología y Salud Pública (CIBERESP) (Barcelona, Spain)

**Corresponding Author’s contact info:**

Sergio Moral, MD, PhD

Cardiology Department, Hospital Universitari Doctor Josep Trueta Avenida França, S/N, 17007, Girona, Spain

Phone: +34 972940200; FAX: +34 972 940 270 E-mail: [moral.sergio@yahoo.es](mailto:moral.sergio@yahoo.es)

**Potential conflicts of interest:** none.

**Table A1**. Search strategy used in the current systematic review and meta-analysis.

| **Queries**  (between January 2007 and May 2019) | **Total Studies**  (including duplicates) |
| --- | --- |
| “Right-sided infective endocarditis” | 319 |
| “Right-sided infective endocarditis” AND “prognosis” | 81 |
| “Right-sided infective endocarditis” AND “intravenous drug user” | 31 |
| “Right-sided infective endocarditis” AND “non intravenous drug user” | 10 |
| “Right-sided infective endocarditis” AND “non drug user” | 12 |
| “Right-sided infective endocarditis” AND “cardiac devices” | 43 |
| “Right-sided infective endocarditis” AND “without association” | 13 |
| “Right-heart infective endocarditis” | 46 |
| “Right-sided infective endocarditis” AND “haemodialysis” | 12 |
| “Right-sided infective endocarditis” AND “central venous catheter” | 6 |
| **Sum of studies evaluated for each category** | **573** |

**Appendix A1**. Modified Newcastle-Ottawa Quality Assessment Scale for Cohort Studies.

**NEWCASTLE - OTTAWA QUALITY ASSESSMENT SCALE**

**COHORT STUDIES**

Note: A study can be awarded a maximum of one star for each numbered item within the Selection and Outcome categories. A maximum of two stars can be given for Comparability. This modified scale allows a maximum of nine (9) stars per study.

**Selection**

1) Representativeness of NODID right-sided infective endocarditis (RSIE) not associated to cardiac devices or intravenous drug users

a) truly representative of the average RSIE in the community **🟑**

b) somewhat representative of the average RSIE in the community **🟑**

c) selected group of users eg nurses, volunteers

d) no description of the derivation of the cohort

2) Selection of non-NODID RSIE cohort

a) drawn from the same community as the exposed cohort **🟑**

b) drawn from a different source

c) no description of the derivation of the non exposed cohort

3) Ascertainment of NODID RSIE

a) secure record (surgical records or imaging technique confirmation) **🟑**

b) medical records without imaging or surgical information **🟑**

c) written self report

d) no description

4) Demonstration that outcome of interest (endocarditis-related event) was not present at start of study

a) yes **🟑**

b) no

**Comparability**

1) Comparability of cohorts on the basis of the design or analysis: a maximum of two stars were awarded if the study controlled for important confounders either at the stage of study design (restriction/stratification) or analysis (adjustment). These included:

a) study controls for NODID RSIE outcomes in RSIE **🟑**

b) study controls for other well-defined RSIE groups (such as those associated to cardiac devices or intravenous drug users) outcomes in RSIE **🟑**

**Outcome**

1) Assessment of outcome

a) independent blind assessment of all outcomes using standard protocols**🟑**

b) record linkage **🟑**

c) self report

d) no description

2) Was follow-up long enough for outcomes to occur

a) yes **🟑**

b) no

3) Adequacy of follow up of cohorts

a) complete follow up - all subjects accounted for **🟑**

b) subjects lost to follow up unlikely to introduce bias - small number lost <20% follow up, or description provided of those lost **🟑**

c) follow up rate < 80% and no description of those lost

d) no statement

**Table A2. Clinical complications of patients with NODID vs non-NODID RSIE (divided in those with devices or IVDU) during hospitalization and after discharge.**

| **Variables** | **RSIE with devices** | **RSIE in IVDU** | **NODID RSIE** | **p** |
| --- | --- | --- | --- | --- |
| **CLINICAL OUTCOME DURING HOSPITALIZATION** | | | | |
| **(n=57)** | **(n=24)** | **(n=11)** | **(n=22)** |  |
| **In-hospital mortality and/or open-heart surgery, n (%)** | **1 (4)** | **1 (9)** | **9 (41)** | **0.004** |
| **In-hospital mortality, n (%)** | **1 (4)** | **0 (0)** | **5 (23)** | **0.039** |
| **Open-heart surgery, n (%)** | **0 (0)** | **1 (9)** | **4 (18)** | **0.042** |
| **Pulmonary embolism, n (%)** | **6 (25)** | **11 (100)** | **11 (50)** | **<0.001** |
| **Systemic embolism, n (%)** | **0 (0)** | **3 (27)** | **0 (0)** | **0.005** |
| **CLINICAL OUTCOME AFTER DISCHARGE** | | | | |
| **n= 51** | **n= 23** | **n=11** | **n=17** |  |
| **Mortality for any cause of death, n (%)** | 6 (26) | 1 (9) | 6 (35) | 0.298 |
| **Mortality for oncologic pathologies, n (%)** | 0 (0) | 0 (0) | 2 (12) | 0.102 |
| **Mortality for respiratory pathologies, n (%)** | 1 (4) | 0 (0) | 1 (6) | 0.594 |
| **Mortality for cardiac pathologies, n (%)** | 1 (4) | 0 (0) | 2 (12) | 0.320 |
| **Mortality for right heart failure, n (%)** | 0 (0) | 0 (0) | 1 (6) | 0.327 |
| **Mortality for other causes, n (%)** | 4 (17) | 1 (9) | 1 (6) | 0.501 |
| **Relapse, n (%)** | 0 (0) | 1 (9) | 0 (0) | 0.208 |

IVDU = Intravenous drug users; RSIE: right-sided infective endocarditis

**Table A3.** Clinical complications during hospitalization of patients with RSIE associated with cardiac devices vs NODID RSIE divided in three groups: catheter carriers, associated with congenital heart diseases and cases without these features.

| **Variables** | **RSIE with cardiac devices** | **NODID RSIE** | | | **p** |
| --- | --- | --- | --- | --- | --- |
|  |  | **RSIE with catheters** | **RSIE with congenital heart diseases** | **RSIE without these features** |  |
| **CLINICAL OUTCOME DURING HOSPITALIZATION** | | | | | |
| **(n=46)** | **(n=24)** | **(n=6)** | **(n=4)** | **(n=12)** |  |
| **In-hospital mortality and/or open-heart surgery, n (%)** | **1 (4)** | **2 (33)** | **2 (50)** | **5 (42)** | **0.016** |
| **In-hospital mortality, n (%)** | **1 (4)** | **1 (17)** | **0 (0)** | **4 (33)** | **0.085** |
| **Open-heart surgery, n (%)** | **0 (0)** | **1 (17)** | **2 (50)** | **1 (8)** | **0.025** |
| **Pulmonary embolism, n (%)** | **6 (25)** | **3 (50)** | **2 (50)** | **6 (50)** | **0.375** |
| **Systemic embolism, n (%)** | **0 (0)** | **0 (0)** | **0 (0)** | **0 (0)** | **---** |

**Table A4.** Selected characteristics of the 4 studies included in this systematic

review and meta-analysis.

| **Source** | **Location** | **Type** | **Sample size of RSIE** | **Men** | **Mean**  **age** | **Incidence of NODID RSIE** | **In-hospital mortality of NODID RSIE** | **Open-heart surgery of NODID RSIE** | **NOS** |
| --- | --- | --- | --- | --- | --- | --- | --- | --- | --- |
| Ortiz C et al 2014 (11) | Spain | Prospective cohort | 121p | 74% | 55y | 20p  (17%) | 6p  (30%) | 8p  (40%) | 9 |
| Mi MY et al, 2016 (10) | United States | Retrospective cohort | 105p | 43% | 45y | 29p  (28%) | 8p  (28%) | -- | 7 |
| Georges H et al 2018 (13) | France | Retrospective cohort | 37p | 54% | 48y | 25p  (68%) | 6p  (24%) | -- | 7 |
| Current series, 2019 | Spain | Retrospective cohort | 57p | 68% | 59y | 22p  (39%) | 5p  (23%) | 4p  (18%) | 9 |

NOS: Newcastle-Ottawa score; RSIE: right-sided infective endocarditis; p=patients; y=years

**Table A5.** Newcastle‐Ottawa Risk of Bias Assessment.

|  | **S1** | **S2** | **S3** | **S4** | **C1** | **C2** | **O1** | **O2** | **O3** | **Total** |
| --- | --- | --- | --- | --- | --- | --- | --- | --- | --- | --- |
| Ortiz C (11), 2014 | 1 | 1 | 1 | 1 | 1 | 1 | 1 | 1 | 1 | 9 |
| Mi MY (10), 2016 | 0 | 1 | 1 | 1 | 1 | 0 | 1 | 1 | 1 | 7 |
| Georges H (13), 2018 | 0 | 1 | 1 | 1 | 1 | 0 | 1 | 1 | 1 | 7 |
| Current series, 2019 | 1 | 1 | 1 | 1 | 1 | 1 | 1 | 1 | 1 | 9 |
| **Mean values** | 2/4 | 4/4 | 4/4 | 4/4 | 4/4 | 2/4 | 4/4 | 4/4 | 4/4 | 8 |

* For an explanation of the individual items on selection (S), comparability (C) and outcome (O), see the provided NOS. Maximal NOS score is 9.
